# Mercurials in Dysentery

**Published:** 1852-10

**Authors:** S. B. Hunt

**Affiliations:** Mendon, N. Y.


### ART. V. — "*Mercurials in Dysentery.*" By S. B. Hunt, M. D., Mendon, N. Y.

You will, I am assured, pardon me for again trespassing upon your kindness. The subject of "Mercurials in Dysentery" is, at present, so far as the pages of your Journal are concerned, left in a shape which I do not like. My communication in the September number was brief and hastily written' and the propositions which I advanced were too ill digested to withstand your searching criticism. I am, however, rather antagonistic in my nature, and am, as yet, unwilling to abandon my ground.

The question at issue is whether mercurials are necessary or proper in the treatment of dysentery. Now much depends upon what we call dysentery. If you rule down the term in its application to simple, unmixed, acute dysentery, I acknowledge myself at once *hors du combat.* But inasmuch as we of the country see but little of this simple dysentery, and that little easily controlled by opiates and rest, we do not, when speaking of dysentery, have this form of disease in our minds, but rather a disease wherein the symptoms of unmixed dysentery are present in great force, but accompanied and modified by a train of symptoms, which add greatly to the difficulty of its management. These symptoms are those to which universal consent has given the name — *bilious.* Do not accuse me of having shifted my ground. My previous article had reference, mainly, to the Portage Epidemic of 1848. My precise ground on this point of etiology is this: I suppose that portal congestion, pre-existing in a constitution exposed to extreme heat, or sudden changes from extreme heat to cold, will, in the majority of cases, determine the production of dysentery in preference to diarrhœa. Common crapulous diarrhœa may result from the irritant nature of the ingesta, or it may depend upon an irritable condition of the intestinal mucous membrane produced by the common causes of bowel complaint, mentioned above. This irritable condition of the bowels may run into follicular enteritis, and in constitutions enfeebled by city residence, and less frequently in those removed from that influence, may even go farther into that pronounced form of muco-enteritis which we call dysentery, and this may and does happen without any portal congestion. But it will happen far more frequently when portal congestion exists.

The term "*bilious*," it is true, has been wofully misapplied, but it still has, in my mind, a single definite meaning — confined to that state of the liver where, be the ultimate cause what it may, the bile is not properly excreted, and manifests its presence in the circulation by discoloration of the conjunctiva and skin, and frequently the urine and sweat — by a whitish tongue — somnolency at noon-day — headache — langour — dull pain in right side, nausea and loss of appetite. Much as we deplore the indolence and want of research which would make the liver the common progenitor of all disease, we should no less avoid that contrary error, which would ignore the very existence of a liver, and confine its disturbances in their effects to the organ itself, forgetting that through it pours the whole vast tide of the abdominal circulation; of which it stands the headgate and governor.

Should you insist, as you intimate you might do, upon my producing *positive* evidence as to the malarial origin of the disease in question, I might find it difficult to convince you. It is extremely difficult to reason from a class of facts so intangible as the ultimate causes of disease. A large class of minds in their zeal for making medicine an exact science, rule out everything which would not be evidence in a court of justice. In regard to this particular epidemic at Portage, I can only state my firm conviction that malarial causes were at work. I have, in my previous article, supplied you with the medical topography of the township — I will only add that at the Deep Cut of the Genesee Valley Canal, the same causes were at work upon a grander scale, as those described by Prof. Hamilton, as, in his opinion, the exciting causes of cholera in Ellicott street, Buffalo. I confess that I had supposed it to be a settled point that malarial causes produce hepatic disease; no tissue of reasoning can stand up against the well known fact, that hepatic diseases have their favorite haunts in low, warm situations, along the banks of sluggish streams, upon the flats and bottom lands adjoining great rivers, and in the forest newly laid open by the axe of the pioneer to the solar rays. Wherever I have seen fever and ague, I have seen accompanying hepatic disease, and the universally received nomenclature, which has given to miasmatic disorders the familiar generic term of bilious disease, is an evidence of a deep fixed conviction in the medical mind of the existence of such a thing as a bilious state of the system.

In the summer of 1847 I was attending the family of B. W. French, living on the verge of Mendville flats, on the Genesee river. Six or seven of ten families were down with dysentery at a time. The house stood at a slight elevation above the flats, w[illegible]h were every morning covered by a dense fog, the smell of which was positively *rotten.* This fog was stratiform, and I have frequently, in climbing a slight knoll, found myself above its level, and seen it stretched like a lake across the wide fertile valley, while here and there an isolated tree or the roof of a barn, lay like an island on its surface.

I had the idea at the time, that this fog was surcharged with miasma, and that to it Mr. French and his neighbors were indebted for their dysentery which prevailed through the valley, while others of them were shaking with the ague from the same cause. At any rate they were decidedly *bilious*, having all those symptoms which I have enumerated as peculiar to that condition, and which they possessed in common with their fever and ague neighbors. People living on the hill-side above the level of this paludal exhalation were healthy. Supposing the exhalation from the flats to be the cause of the disease, we would expect patients nightly under the influence of that cause to be difficult of cure. Such was the case. They were the most intractable cases I had that season, and only recovered when I ordered the sick to the upper part of the house, and the doors and windows to be kept closed until such an hour in the morning as the fog was dissipated. Then they got well with as much rapidity as the cholera disappeared from Ellicott street, when that ditch was closed.

My good old preceptor, Prof. Trowbridge, said, that no one took fever and ague in the daytime, and it was his practice when spending the night with his patients in the malarial district on the Grand River below Painesville, Ohio, to sleep in an upper room, and to this he attributed his entire immunity from malarial disease.

And now about the inflammatory nature of the disease. I certainly had no intention of denying the existence of ulceration, deposits of lymph, etc. I have seen some post-mortem appearances in dysentery. Instead of the term "purely inflammatory," I should have said that the disease, as it has more usually presented itself to my notice, is not *simply* inflammatory in its nature, i. e., that the inflammation is so modified by its causes, as to present different symptoms from any that could result from a simple muco-enteritis. The grade of fever was low, in many cases a periodical tendency to chill could be detected; in many, too, the patient was completely prostrated at the outset of the disease, and, in a state more resembling congestive fever than any other pathological condition. I meant to convey the idea that in cases depending as I believed on paludal origin, the disease was not a simple muco-enteritis yielding to opium, entire rest, and the abstraction of blood, but an inflammation modified by the causes which gave it birth, and requiring some remedy reaching beyond the locale of the disease, to the causes in which it originated. That such a form of dysentery exists as you describe, I cannot doubt, for I have seen it and allude to the opium treatment in my article as successful in those cases. But I think it is in cities where they mostly obtain.

In this lengthy discussion of etiology and pathology it has been my purpose to keep an eye to the main question at issue, viz., the propriety of the exhibition of calomel.

In coming to the discussion of therapeutical effects we tread at once on more satisfactory ground. We administer many drugs empirically, without any pathological reasons therefor. When we derive benefit from the exhibition of a drug, it is a matter which our own eyes can see. When we refuse to administer it from preconceived notions as to the pathology of a disease, we are working in the dark, because our notions of pathology are much more liable to error than is an opinion as to the therapeutic effects of a drug derived from experience. No one could argue *a priori* that capsicum would be a good gargle for a sore throat, yet experience has taught us its value.

A difference of opinion as to the point in question is no new thing. Writers are so divided that a collection of authorities would yield no light. Calomel has long been used in the whole class of bowel diseases, and it seems still to have as strong a hold as ever. In dysentery the modes of its administration have varied. I have myself usually given it in doses of three grs., combined with Dover's powder in ten grain doses for three or four doses in succession, and have followed it with some mild cathartic. I have known it given in full cathartic dose at the outset of the disease, and have the word of respectable physicians as to its efficacy. This is, I am informed, a very common practice in those malarial regions in Maryland and eastern Pennsylvania, where dysentery is a formidable yearly visitant. It is also sometimes given with a view to its sedative effect, in doses of from ℈j. to ʒss. half hourly. It is claimed that this practice has been attended with unrivaled success in tropical dysentery. Of these latter modes of administration I have no personal knowledge. Of the first mentioned manner of exhibition I have an experimental knowledge, which is to me satisfactory. In 1846, when my acquaintance with the disease commenced, I was in very limited practice, and saw but few cases. There were no deaths. In 1847 I attended about twenty cases, but I do not know the exact number. There were no deaths. In 1848 I attended 130 cases, and had six deaths; one of these was a feeble old man of over seventy years, the others were children. I was, at the close of the epidemic of the opinion, that had I been more thorough in the administration of calomel, I might have lost fewer cases. In 1849 I attended twenty-eight cases; of these one was fatal, a man eighty years of age.

Since my removal to my present location, I have seen less of the disease by far, than when at Portage. I have, however, carefully watched the effect of the remedy, and am more than ever convinced of its efficacy. I think that even in the medium doses which I administer, it has something of that sedative effect attributed to the heroic dose. I always consider that when I have once obtained a fœcal evacuation the disease is under control. Aside from its effect upon the biliary secretions, it assists the sudorifics with which it is combined in directing to the surface, it has its usual specific action upon the inflammation, and what is very important, it prepares the way for a cathartic. There is in many severe cases of dysentery a stricture at the caput coli, which prevents the passage of anything from the upper bowel, save in quantities so small as to add to the irritation at the seat of the disease. Accumulations of fœcal matter and vitiated secretions form above this stricture, and it is frequently difficult to get cathartics past this point without calomel preceding their administration.

During the Peninsular war, and especially in those disastrous retreats made by the British army, the troops, sleeping upon the ground uncovered, and exposed to all the forms of miasma, were visited by dysentery in its unmanageable form.

In two years and a half the British army in the Peninsula lost no fewer than 4,717 men by this "scourge of armies." Sir James McGrigor, who had the superintendence of the medical department in these campaigns, and in the no less disastrous Walcherean expedition, after opportunities for observation unequaled in the history of the disease, recommended to the hospital surgeons a treatment, in which calomel, followed by a saline cathartic, figured as the leading prescription. It was given upon alternate days, the bowels being closed with Dover's powder immediately after the operation of the cathartic. I believe that this will still be found a satisfactory treatment in all cases where portal congestion exists, and I have already expressed my belief as to the frequency of these cases.

There has been much dispute as to the propriety of cathartics. I consider them as an important adjuvant. Constipation is one of the most constant concomitants of dysentery. If we give no cathartics we have, in spite of opiates, a constant dribbling of fœcal matter from above over the inflamed surface. *With* a cathartic we sweep them out at once, and are thus enabled to keep the bowels quiet with opiates after the operation. The cathartic chosen should be one quick and thorough in its operation. I prefer the Rochelle salt to any other.

The use of astringents I have already objected to. They are unphilosophical in every point of view, unless we regard the disease as a passive hæmorrhage. *En passant, does* opium in the dose which you propose "lock up the secretions of the liver" or any other organ? In a case of persistent spasm of the intestines under treatment last week, I was obliged to keep the patient under the most powerful doses of opium. I gave him twelve grs. of sulph. morph. in twelve hours. Afterward I gave sixty drops of laudanum, hourly, for twenty-four hours; and after that forty-five drops, hourly, for another twenty-four hours. During this time I procured, by enema, free evacuations of slime and mucus, gradually changing as the disease declined, to fœcal matter. There was certainly no deficiency of secretion in this case, for large quantities of bile were present in the stools.

I have only one thing more to say as to the treatment of dysentery, and that is about injections. I have found the cold water enema of far greater service than the more usual mixture of starch and laudanum given warm.

In conclusion, I beg that you may not rank me as an indiscriminate advocate of calomel. I believe I use less than the average of practitioners, and have even, the past summer, treated hypertrophied liver with the alkaline carbonates, and without calomel, successfully.

